# Cases of Intermittent and Remittent Fever, Treated with Chloroform, Internally

**Published:** 1867-10

**Authors:** Geo. F. Brickett

**Affiliations:** Chicago


ARTICLE XLV.

### CASES OF INTERMITTENT AND REMITTENT FEVER, TREATED WITH CHLOROFORM, INTERNALLY.

By GEO. F. BRICKETT, M.D., of Chicago.

Case I. Mrs. C., native of Georgia; resident of St. Helena Island, S.C.; *æt.* 25; has had repeated attacks of intermittent fever for the past four years; was called to visit her July 28, 1865, and found her suffering with a decided chill, and vomiting every few moments. She informed me that she had had a chill the day before, at the same hour, 8 A.M., which lasted about fifteen minutes. I ordered one fluid drachm of chloroform, (undiluted,) to be administered at once. She soon fell into a calm slumber, of one hour and twenty-five minutes duration. She expressed herself to me, on awakening, as feeling so well, that I gave her permission to leave her room that day. I made her a visit next day, one hour after the time of the expected chill, and finding her well, I did not leave any prescription; I watched the case several days, but found no treatment required. From that date she has had no treatment, and has had no symptoms of malarial fever. On questioning her as to the effects experienced from the chloroform, she said that immediately after taking the medicine, she felt a warm sensation in the stomach, and a pecular numbness pervading her whole system, but more marked than elsewhere in her hands and feet. These sensations lasted but a few moments, and were succeeded by a calm slumber.

Case II. Lieut. L., U. S. C. T.; *æt.* 24; native of Vermont. At 2 P.M., on the 29th day of July last, was called to see this patient, residing in the same house, and suffering from the same disease as the preceding case. The success attending the administration of the chloroform in Mrs. C.'s case, induced me to suggest the propriety of his taking the same remedy; but he believing it to be a mere matter of experiment with me, and reposing great confidence in quinine, requested that the latter medicine be administered, which was accordingly done. On the 4th of August last, while he was suffering from a severe chill, I was sent for; and he begged me to give him anything that would afford him relief; I at once administered one fluid drachm of chloroform, (undiluted,) with the satisfaction of soon seeing the chill pass off, and the patient fall into a quiet sleep. On awakening, two hours from the time the chloroform was given, a slight febrile reaction followed. At the time of his leaving the Island, (about the 1st of September,) he had had no further treatment, and no symptoms of a return of the malarial disease.

CASE III. Mrs. A.; native of Ireland; *æt.* 30; resident of St. Helena Island, S.C. Visited her on the 16th day of September last, and found a well-marked attack of intermittent fever. Having no chloroform at hand, gave her quinine; she continued taking quinine (4 grs. a day) until the 10th of October, when her supply became exhausted. On the 26th ultimo, she had another attack of the same fever. Being sent for I administered to her, while in a chill, one fluid drachm of chloroform, and ordered to be removed all means of warmth, as blankets, hot bricks, etc. The immediate effect of the remedy was to produce a quiet slumber. After two hours' sleep, she awoke, with only a slight febrile reaction. No other medicine has been given, and up to this time has had no return of the fever.

CASE IV. Mr. C. D., Acting 3d Assistant Engineer, U. S.N.; *æt.* 25; native of Maryland. Afflicted with remittent fever two days before admission into the hospital; a great deal of febrile excitement followed the chill. He was treated with quinine before admission, but not regularly. On his admission I ordered him to take 5 grs. of the sulphate of quinine, three times a day; but with little effect. On the 3d of November, three days after his admission, one fluid drachm of chloroform was given him one hour before the expected chill. After taking it, slept one hour, and awoke greatly relieved. No further treatment was found necessary, and the officer returned to duty four days afterwards.

CASE V. Mr. A. M., chief clerk to office of M. E. D. S. C.; native of New York; *æt.* 40. Called to this case, Nov. 2d, and saw him while in a chill. He informed me that he had had a slight chill the day before, at the same hour, 2 P.M.; found him enveloped in blankets, and drinking hot whiskey punch; ordered punch to be stopped, blankets to be removed, and one fluid drachm of chloroform to be administered. The same effect seen as in previous cases, viz.: slumber, and feeling, on awakening, freshened and restored. In this case, however, when I called on him next morning, I found to be present some slight dysenteric symptoms, which were readily removed by simple remedies, and he was returned to duty within a week. He has not had a chill since the chloroform was given.

CASE VI. Lieut. Q., U. S. C. T.; native of Wisconsin; *æt.* 25; station on St. Helena Island. Taken sick on the 2d of November. Found him in a most severe chill; immediately gave him a fluid drachm of chloroform; on going to see him next morning, was surprised to see him busily employed at some out-door duty, superintending the construction of quarters. He expressed himself as feeling "as well as ever." I would state, that four weeks previous to this last attack, this officer was sent to an army hospital, suffering with a most severe form of malarial fever, and was, on this account, absent three weeks from his post. On his return to this Island he was again attacked, when I administered chloroform, with the above happy result. Since the last attack no medicine has been given, and he has been in the enjoyment of perfect health.

*Commentary.*—I would remark, that in case I., the chloroform was administered with the intention of relieving the emesis, without thinking it would be found efficacious to the removal of the disease. The happy result that then followed was, of course, entirely unexpected.

The appearance in the October number of the *American Journal*, of Dr. MERRILL'S excellent article, gave me confidence in the remedy, and I resolved to test it in every suitable case. In each of my cases, in previous attacks, it had been found necessary to administer quinine for several successive days, before any decided relief was obtained.

Without going to the extreme, of proposing chloroform as a "specific" in these diseases, I would merely call the attention of the profession to this remedy, and I would earnestly solicit further experiments with it.

I deem it just to state that my cases were all frank and open, and without any but the ordinary complications incident to these diseases.

The highly beneficial effect of the administration of chloroform, *without the subsequent use of quinia*, in the cases in which I have given, it justifies me, I think, in placing my experience before the medical public. If these few cases should be considered sufficiently important to attract the attention of the profession, and thereby induce others to experiment with this remedy, I shall consider myself amply rewarded.

